# Congenital Anterior Urethral Diverticulum in a Male Teenager: A Case Report and Review of the Literature

**DOI:** 10.1155/2011/738638

**Published:** 2011-09-28

**Authors:** Sadat Haider Quoraishi, Faisal Khan, Dler Besarani, Krishna Patil

**Affiliations:** Department of Urology, Ashford and St. Peter's Hospitals NHS Trust, Guildford Road, Chertsey, Surrey, KT16 0PZ, UK

## Abstract

We present the case of a 13-year-old boy with a congenital anterior urethral diverticulum. This is a rare condition in males which can lead to obstructive lower urinary tract symptoms and urosepsis. Diagnosis is by urethroscopy and radiological imaging. Surgical treatment can be open or endoscopic. Long-term followup is required to check for reoccurrence of the obstruction.

## 1. Case Report

A 13-year-old boy presented with a two-month history of dysuria, suprapubic discomfort, and poor urinary flow. There was no other significant history. Examination revealed no abnormalities.

**Figure 1:**
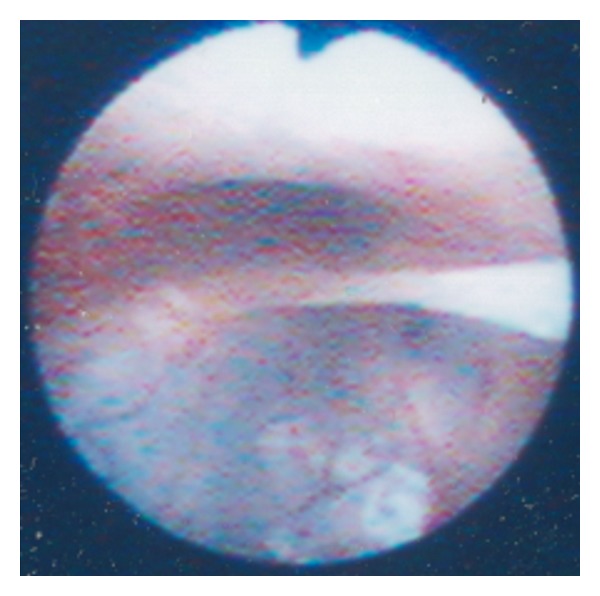
Cystoscopic view of the anterior urethra, showing the true lumen and the urethral diverticulum. A band of tissue separates the two, and this was subsequently incised.

Urine culture, ultrasound of the urinary tract, and magnetic resonance imaging of the spine, pelvis, and urethra were all normal. However, the average urinary flow rate was only 2.1 mL/s. Rigid cystoscopy revealed an anterior urethral diverticulum (Figure 1). A retrograde urethrogram showed persistent dilatation of the bulbar urethra.

The patient underwent three urethroscopic incisions of the neck of the diverticulum over two years. After the first two procedures there was a temporary improvement in the urinary flow rate, followed by a worsening of symptoms due to scar tissue. Now the average flow rate is 12.2 mL/s. The patient believes that his urinary flow has returned to normal. The plan is to monitor the urinary flow rate in the out-patient clinic and to repeat the diverticulum neck incision if the flow rate worsens.

## 2. Discussion

Urethral diverticula belong to a spectrum of obstructive anterior urethral conditions that include anterior urethral valves. Some authors do not distinguish between the conditions while others do [1]. The common feature is a blind-ending outpouching of the urethra. An anterior urethral valve is completely contained within the corpus spongiosum whereas a diverticulum breaches through the corpus spongiosum into surrounding tissue. We have not made the distinction in this paper as the management is the same for both. A flap can develop at the lip of the diverticulum which rises into the urethral lumen as the diverticulum fills with urine, causing an obstruction. Congenital urethral diverticula in males are rare, and patients can present with poor urinary flow, postmicturition dribbling, urinary tract infections, or penile ballooning in the neonatal and infancy period [2, 3], although later presentations into the teenage years have been known. The aetiology is unknown but theories include a ruptured syringocoele, cystic dilatation of the periurethral glands, and incomplete hypospadias [4].

Retrograde urethtography and voiding cystourethtography may be used to image the male urethra [3, 5]. Urethroscopy will reveal an appearance of the urethral lumen splitting into two—one passage is the true lumen while the other is the diverticulum, with the flap or distal lip of the diverticulum dividing the two.

Management of the lesion can be endoscopic or open [1, 6]. We adopted an endoscopic approach with incision of the lip of the diverticulum, although, as the diverticulum pouch still exists, it can again develop a flap, requiring repeat procedures. Subsequent scar tissue formation may result in a urethral stricture which, like the diverticulum, can cause poor urinary flow, recurrent urinary tract infections, and bladder stones. Therefore regular endoscopic surveillance and correction may be required. An open approach (patch graft urethroplasty) can also be used to excise the diverticulum permanently and reconstruct the urethra, giving it a more uniform calibre; however there is the risk of urethrocutaneous fistula formation. If the patient has urosepsis or obstructive nephropathy, a temporary urinary diversion by way of suprapubic catheterisation can be employed before surgical treatment [1].
